# Association of the nasopharyngeal carcinoma and the subsequent open glaucoma development: a nationwide cohort study

**DOI:** 10.7150/ijms.80837

**Published:** 2023-04-17

**Authors:** Wei-Yang Lu, Chiao-Wen Lin, Chung-Han Hsin, Chia-Yi Lee, Jing-Yang Huang, Shun-Fa Yang, Hung-Yu Lin

**Affiliations:** 1 Institute of Medicine, Chung Shan Medical University, Taichung, Taiwan; 2 Department of Ophthalmology, Changhua Christian Hospital, Changhua, Taiwan; 3 Department of Optometry, Chung Shan Medical University, Taichung, Taiwan; 4 Department of Post-Baccalaureate Medicine, College of Medicine, National Chung Hsing University, Taichung, Taiwan; 5 Institute of Oral Sciences, Chung Shan Medical University, Taichung, Taiwan; 6 Department of Otolaryngology, Chung Shan Medical University Hospital, Taichung, Taiwan; 7 School of Medicine, Chung Shan Medical University, Taichung, Taiwan; 8 Nobel Eye Institute, Taipei, Taiwan; 9 Department of Ophthalmology, Jen-Ai Hospital Dali Branch, Taichung, Taiwan;; 10 Department of Medical Research, Chung Shan Medical University Hospital, Taichung, Taiwan; 11 Department of Ophthalmology, Show Chwan Memorial Hospital, Changhua, Taiwan

**Keywords:** nasopharyngeal carcinoma, open angle glaucoma, age, epidemiology

## Abstract

This study aimed to investigate the possible association between nasopharyngeal carcinoma (NPC) and following open angle glaucoma (OAG). A retrospective research applying the National Health Insurance Research Database (NHIRD) of Taiwan was conducted with a follow up period from January 1, 2000 to December 31, 2016. There were 4184 and 16736 participants that selected and categorized into the NPC and non-NPC groups after exclusion. The major outcome of our study was the development of OAG according to diagnostic codes, exam and managements. The Cox proportional hazard regression was employed to estimate the adjusted hazard ratio (aHR) and 95% confidence interval (CI) of OAG between the two groups. In this study, a numbers of 151 and 513 OAG episodes occurred in the NPC and non-NPC groups and the NPC population showed a significantly higher incidence of OAG compared to the non-NPC population in multivariable analysis (aHR: 1.293, 95% CI: 1.077-1.551, p = 0.0057). Besides, the cumulative probability of OAG was significantly higher in the NPC group than that in the non-NPC population (p = 0.0041). About other risk factor for OAG, age older than 40 years old, diabetes mellitus and persistent steroid usage were related to OAG occurrence (all p < 0.05). In conclusion, the NPC may be an independent risk factor of following OAG development.

## Introduction

Nasopharyngeal carcinoma (NPC) is an epithelial carcinoma that grows at the nasopharyngeal region 1. East and Southeast Asian countries showed a higher incidence of NPC where the prevalence of NPC is 3.0 per 100000 residences in the China area 2, 3. Cigarette consumption, human papillomavirus infection and the Epstein-Barr virus infection are well-known risk factors for the occurrence of NPC 1, 3, 4. The choices of treatments for the NPC involve chemotherapy and radiotherapy and the recurrence of NPC is not uncommon 5, 6. The mean survival interval of late-stage NPC patients were near 3 years which could be further shortened if the patient was under the divorce status 7, 8.

NPC in the advanced stage and the radiotherapy during the NPC treatment could damage nearby regions like the orbital and cervical area 9. Lymphadenopathy and cervical mass are common clinical features in NPC progression 9. Additionally, chronic rhinosinusitis has been found frequently in the individuals diagnosed with NPC 10. The occurrence of NPC and subsequent radiotherapy is associated with auditory system disorder which includes sensorineural hearing loss, otitis media and ear effusion 9, 11. Except for the nasal and auditory complications, neurological complications such as meningitis, swallowing difficulty, cavernous sinus thrombosis, skull base osteoradionecrosis and central nervous system defects have been reported in previous literatures after NPC and the radiotherapy management 12, 13.

Ophthalmic complications in the individuals with NPC have been demonstrated in the previous researches 14. The orbit is the most common region of ophthalmic involvement in the patients with NPC which account for nearly 50 % of NPC cases with ophthalmic complications 14. In addition, proptosis and eyelid swelling were also observed in previous research 15. However, there are few studies evaluating the relationship between the NPC and open angle glaucoma (OAG). Because the OAG is a type of neuropathy and optic neuropathy has been found in patients with NPC 15, 16, the existence of NPC may be associated with following OAG which need additional investigation.

Accordingly, the purpose of our study is to analyze the possible correlation between NPC and the consecutive OAG via the National Health Insurance Research Database (NHIRD) of Taiwan. Age, sex and several systemic comorbidities were also enrolled in the analysis model.

## Materials and Methods

### Data source

The procedure in our study adhered to the declaration of Helsinki in 1964 and the late amendments. Our study was also approved by both the Institutional Review Board of Chung Shan Medical University Hospital (Project identification code: CS1-20108), and the National Health Insurance Administration. In addition, the need of informed consent was waived by the two institutions listed above. The NHIRD in Taiwan holds the claimed data of the health insurance program and the individuals included in the NHIRD is approximately 23 million Taiwanese from January 1, 2000 to December 31, 2016. The applicable information in NHIRD carries the International Classification of Diseases, Ninth Revision (ICD-9) diagnostic code, age, education level, sex, medical department type, place of inhabitant, image exam, laboratory exam, procedure and surgery codes, and international ATC codes for medicines. We utilized the longitudinal health insurance database (LHID) 2000 version, a sub-database developed from NHIRD, for the data access and statistical analyses in the current study. The LHID 2000 holds about two million patients who were randomly chosen by the automated software from the NHIRD at 2000, and all the information in NHIRD are also accessible in the LHID 2000. Besides, the participants in the LHID 2000 were traced with same interval as in the NHIRD which expresses a period from January 1, 2000 to December 31, 2016.

### Patient Selection

In this retrospective cohort study, NPC was defined with the following criteria: (1) receipt of ICD-9 codes of NPC, (2) biopsy and sinoscopy arrangement before or simultaneously with NPC diagnosis, (3) Epstein-Barr virus DNA exam arrangement before or simultaneously with NPC diagnosis, (4) follow up in the NHIRD/LHID 2000 for at least 3 years, and (5) the NPC diagnosis was done by an otorhinolaryngologist. The index date was the date one year after the NPC diagnosis. To elevate the homogeneity of participants in our study, we also administered some exclusion criteria: (1) participants younger than 20 years old, (2) diagnosis of blindness before the index date, (3) the receipt of eyeball removal surgery before index date, (4) diagnosis of ocular tumor before the index date, (5) the receipt of corneal transplantation before the index date, (6) outcome developed before index date, (7) the patient died before index date. After exclusion process, one NPC members were age- and sex-matched to 8 non-NPC members. Then these patients were further matched by propensity-score match (PSM) with demographic data (including sex, age) and systemic co-morbidities (including hypertension, diabetes mellitus (DM), acute myocardial infarction (AMI), stable coronary arterial disease (CAD), cerebrovascular disease, hyperlipidemia, pulmonary diseases, rheumatic disease, kidney disease and persistent steroid usage). The ratio between NPC and non-NPC populations in PSM was 1:4. Finally, 4,184 NPC participants and 16,736 non-NPC members built the NPC and non-NPC groups in our study.

### Major Outcome Measurement

The major outcome in our study was regarded as the incident of OAG. To be more specific, only the OAG that accomplished these condition was considered as outcome achievement: (1) the emergence of OAG-related ICD-9 diagnostic codes, (2) the performance of fundoscopic exam, optical coherence tomography or visual field exam before or simultaneously with the OAG diagnosis, (3) the utilization of oral or topical anti-glaucomatous medications after the diagnosis of OAG, and (4) the OAG diagnosis was established by an ophthalmologist. Noteworthy, only members developed OAG after the index date was regarded as major outcome achievement, and the participants were followed from 0 to up to 15 years.

### Demographic and Co-morbidity Covariates

To reduce the impact of possible confounders of OAG, we adjusted the effect of consecutive factors in the statistical analyses: age, sex, hypertension, DM, AMI, stable CAD, cerebrovascular disease, hyperlipidemia, pulmonary diseases, rheumatic disease, kidney disease and persistent steroid usage. To guarantee the systemic co-morbidities are persistent status rather than a short-term episode, we selected the systemic disease with an interval more than two years and the usage of any form of steroid for more than 6 months in the statistical model. We seek our patients until the achievement of major outcome, drop out from the National Health Insurance Program or to the final of NHIRD/LHID 2000 which represents December 31, 2016.

### Statistical Analysis

The SAS version 9.4 (SAS Institute Inc, NC, USA) was conducted for the analysis in the current study. The absolute standardized difference (ASD) was employed to evaluate the difference of basic characteristics distribution between the NPC group and non-NPC group. ASD value larger than 0.1 was defined as significant difference in our study. Then we employed the Poisson regression for the incidence density of OAG and administered the Cox proportional hazard regression for adjusted hazard ratios (aHR) and its 95% confidence intervals (CI) of OAG between the NPC group and non-NPC group. Moreover, Cox proportional hazard regression in our study employs the impact of demographic data and systemic disorders in statistical analysis. The possible actions of confounders on OAG occurrence were also estimated via Cox proportional hazard regression. We painted the cumulative incidence curve to demonstrate the cumulative incidence of OAG between the NPC group and non-NPC group, and the log rank test was adopted for concluding the significance between the NPC population and non-NPC population. Also, the interaction test was adopted to evaluate the possible effect of age and sex in the subgroup analysis. The descriptive analyses were applied to present the types and numbers of glaucoma treatment in the NPC and non-NPC groups. The anti-glaucomatous medications referred to alpha agonists, carbonic anhydrase inhibitors, beta blockers and prostaglandin analogues, the glaucoma laser therapy referred to laser trabeculoplasty, and the glaucoma surgery referred to trabeculectomy, tube-shunt surgery and cryotherapy. The statistical significance was set as p< 0.05 in our study.

## Results

The basic characteristics between the NPC and on-NPC groups are illustrated in Table 1. About the demographic data, the year of index, age and sex were all similar between the two groups due to the PSM procedure (all ASD < 0.1). Besides, all the systemic co-morbidities showed analogous distribution between the two groups (all ASD < 0.1) (Table 1).

**Figure 1:**
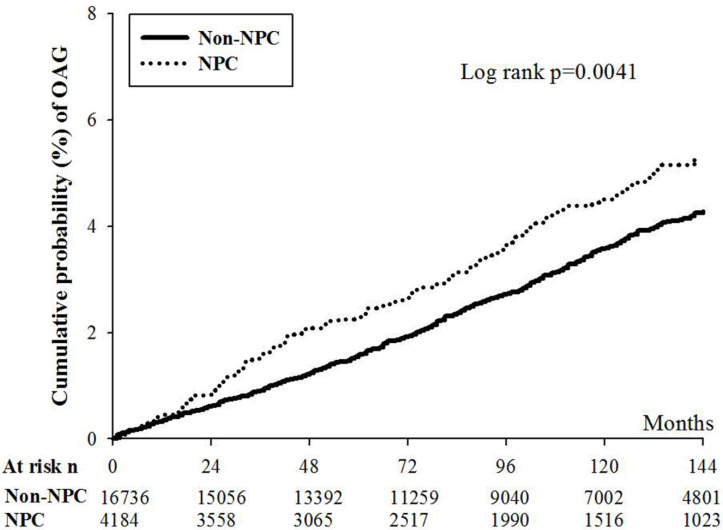
The cumulative incidence curve of open angle glaucoma between the nasal pharyngeal carcinoma and non-nasal pharyngeal carcinoma populations. NPC: nasal pharyngeal carcinoma, OAG: open angle glaucoma, N: number * denotes significant difference between the two groups

There were 151 and 513 OAG episodes in the NPC and non-NPC groups. After adjusting several confounders, the NPC population showed a significantly higher incidence of OAG compared to the non-NPC population (aHR: 1.293, 95% CI: 1.077-1.551, p = 0.0057) (Table 2). Furthermore, the cumulative incidence of OAG was significantly higher in the NPC group than that in the non-NPC group (p = 0.0041) (Figure 1). Concerning the other risk factor for OAG development, age older than 40 years old, the presence of DM and persistent steroid usage were all significant risk factors for OAG occurrence (all p < 0.05). Still, other demographic data and systemic diseases demonstrated insignificantly effect on OAG development (Table 3).

In the subgroup analysis, aHRs of OAG between the male and female NPC patients compared to non-NPC counterpart were similar (p = 0.5018) (Table 4). Moreover, aHRs of OAG among different age population demonstrated insignificant difference (p = 0.6131) (Table 4). Glaucoma treatments for OAG between the two groups are presented in Table 5 and non-NPC group showed numerically higher rates of glaucoma laser therapy and glaucoma surgery.

## Discussion

Briefly, our study demonstrated the higher incidence of OAG in the patients with NPC after adjusting several confounders. Moreover, the cumulative incidence of OAG increased as the duration of NPC become longer. The other independent risk factors for the OAG development included the age older than 40 years old, DM and persistent steroid usage.

Glaucoma would develop under certain specific conditions 17. In general, glaucoma is featured with optic neuropathy and visual field defect 16, and both acute and chronic type of glaucoma have been recorded 18. OAG is a type of glaucoma in which the gonio-angle remains open and the intraocular pressure can be normal or high 17. The etiology of OAG development can be varied while primary or idiopathic OAG accounts for the majority of cases 16. Other predisposing factors of OAG include hypertension and DM according to a review article 19. In addition, the vascular disorders had been proposed as risk factor for OAG development especially in those patients with OAG and normal intraocular pressure 20. In previous studies, patients with stroke had a higher prevalence of OAG than non-stroke individuals 21, and patients with OAG showed a higher chance of following ischemic heart disease 22. Besides, optical coherence tomography angiography revealed decreasing retinal vessel density in patients with high tension glaucoma and normal tension glaucoma 23. On the other side, compressive lesion might be risk factor for OAG development in which the ocular adnexal lymphoma as well as infantile hemangioma and associated OAG have been reported previously 24, 25. Chronic rhinosinusitis contributes to elevated incidence of subsequent glaucoma in a population-based study 26. Regarding NPC, orbital invasion occurred in a large number of patients 14, 27. The mass effect of orbital invasion could cause dislocation of orbital content and related proptosis 15. Furthermore, NPC could affect the orbital apex and lead to compressive optic neuropathy and cavernous sinus defect 28, 29. Because the NPC is frequently associated with orbital invasion which can contribute to mass effect and circulation impairment 14, we proposed that the existence of NPC may exaggerate the probability of glaucoma development especially OAG. This hypothesis was partially supported by the findings of our study.

Concerning the correlation between NPC and subsequent OAG, a previous study demonstrated a case with NPC and glaucoma development 15. In our study, the existence of NPC correlates to a significantly higher incidence of following OAG. To our knowledge, this is a novel finding to show the significant correlation between NPC and OAG with time sequence. In addition, we included several potential risk factors of OAG development including age, metabolic diseases, vascular disorders and steroid application in the multivariable analysis. Consequently, NPC may be an independent risk factor for the OAG development. On the other hand, the cumulative probability of OAG was significantly higher in the NPC population than that in the non-NPC population. This result further indicates the effect of disease interval of NPC on the occurrence of OAG. Since the overall survival rate of NPC within five years was above 50 percent in previous literatures 1, about two percent of these patients may have suffered from OAG according to the Kaplan-Meier curve in our study. This incidence is much higher than the incidence in the non-NPC population in our study and the general incidence of OAG in the study written by Founti et al. 30. Because we only included OAG episodes that developed one year after the diagnosis of NPC, the duration of NPC may be sufficient to trigger following complications and morbidities. However, the exact pathophysiology of this correlation is unknown since both the massive effect and vascular impairment can cause OAG. A further study is warranted to analyse this point. Regarding the epidemiology of OAG, the Chinese population showed a five-year cumulative probability of OAG of 1.3% which was lower than the African population and white race 31. In our study, the five-year cumulative probability of OAG was nearly 2.2 percent in the NPC group and 1.7 percent in the non-NPC group. Consequently, NPC could correlate to numerically higher incidence of OAG comparing to other study with Han ethnicity and the incidence of OAG in our non-NPC population is similar to other Han ethnicity research.

In the subgroup analysis, the male population with NPC showed a significantly higher incidence of OAG compared to the male population without the presence of NPC. On the other hand, the female population with NPC revealed a similar incidence of NPC to the non-NPC women. The role of sex in the development of OAG was conflicting according to previous researches. In a preceding article, the male population owned a higher ratio of OAG episodes compared to female population 32. But another study demonstrated that women would comprise 55 percent of OAG cases 33. Our study showed that the male with NPC may have a slightly higher impact on OAG development than the female with NPC since the interaction test revealed insignificant difference between male and female. NPC patients aged 40-60 years old revealed a higher rate of OAG compared to the non-NPC members aged 40-60 years old. However, other age subgroup did not show significant effect on the OAG development. Because the interaction test among different age subgroups also demonstrated insignificant difference, we speculate that since age is also a well-established risk factor for glaucoma 34, whether the patients with NPC or not may not influence the OAG incidence massively as age increases.

About other risk factors for OAG development in the current study, patients with age older than 40 years old and DM are independent risk factors for OAG development after adjusting multiple covariates of OAG including the NPC. Age is a prominent risk factor for OAG based on the preceding publication 34. In a previous study, the Black ethnicity aged around 40 years old showed a higher OAG risk and the Caucasian ethnicity aged more than 65 years are under higher risk of OAG 35. Another study also demonstrated that age older than 50 years old elevated the risk of OAG at least twofold compared to patients in their 40s' 36. Consequently, it is reasonable that old age served as a significantly predisposing factor of OAG in our study. DM is also a known risk factor for OAG development 37, which possible due to the vascular injury and neovascularization effect. Patients with DM showed a poor outcome of glaucoma surgery compared to the non-DM individuals 38, and neovascular glaucoma is a not-uncommon ocular complication in DM patients 39. Steroids would also lead to glaucoma development significantly 34. The other covariates included in this study did not show prominent impact on OAG development. It is a little strange that hypertension is an insignificant factor for OAG occurrence since a preceding study showed a prominent effect of hypertension on OAG 19. We think maybe the effect of hypertension is overwhelmed by NPC, old age and DM since the previous study did not consider these factors 19.

Some limitations still exist in this study. The claimed-database nature of our study meant that certain critical information could not be accessed, including the severity of confounders, image result of NPC, grade of NPC, treatment program of NPC, degree of orbital involvement in patients with NPC, results of glaucoma-related exam, intraocular pressure of OAG, medications as well as procedures employed in OAG. Secondly, the ICD-9 diagnostic code “unspecific glaucoma” included some individuals with OAG in clinical practice due to convenience. As a consequence, numbers of OAG may be underestimated in our study and influence the statistical outcomes. Additionally, radiotherapy has been applied in more than 60 percent of our patients which could lead to orbital disorder, optic neuropathy and glaucoma which could also related to the OAG occurrence 40, 41. Thus, it is hard to recognize whether the etiology of OAG in our study is due to NPC itself or the radiotherapy for NPC. Finally, there were only 151 episodes of OAG in the NPC population with marginal lower limit of 95% CI which may result in statistical variation, and the generalizability of our study was limited because only Taiwanese were included.

In conclusion, the existence of NPC is significantly correlated to the subsequent OAG after adjusting multiple covariates. Furthermore, the incidence of OAG elevated significantly as the disease interval of NPC increases. A further large-scale prospective study to reveal whether the presence of NPC will alter the prognosis of OAG is mandatory.

## Figures and Tables

**Table 1:** Characteristics among nasopharyngeal carcinoma group and control group after propensity score matching

Character	Non-NPC (N= 16736)	NPC (N= 4184)	ASD
Year of index			0.0000
2001-2005	8010 (47.86%)	2020 (48.28%)	
2006-2010	5168 (30.88%)	1305 (31.19%)	
2011-2015	3558 (21.26%)	859 (20.53%)	
Sex			0.0244
Female	5952 (35.56%)	1537 (36.74%)	
Male	10784 (64.44%)	2647 (63.26%)	
Age			0.0567
20-30	1349 (8.06%)	326 (7.79%)	
30-40	2914 (17.41%)	687 (16.42%)	
40-50	4485 (26.80%)	1100 (26.29%)	
50-60	4363 (26.07%)	1078 (25.76%)	
60-70	2285 (13.65%)	607 (14.51%)	
70-80	1032 (6.17%)	300 (7.17%)	
80-100	308 (1.84%)	86 (2.06%)	
Co-morbidities			
Hypertension	3148 (18.81%)	851 (20.34%)	0.0386
DM	1567 (9.36%)	407 (9.73%)	0.0124
AMI	19 (0.11%)	5 (0.12%)	0.0018
Stable CAD	683 (4.08%)	210 (5.02%)	0.0450
Hyperlipidemia	1529 (9.14%)	424 (10.13%)	0.0338
Cerebrovascular disease	752 (4.49%)	201 (4.80%)	0.0148
Pulmonary diseases	1348 (8.05%)	456 (10.90%)	0.0972
Rheumatic disease	132 (0.79%)	46 (1.10%)	0.0321
Kidney disease	420 (2.51%)	111 (2.65%)	0.0090
Persistent steroid usage	1737 (10.38)	471 (11.25)	0.0580

NPC: nasopharyngeal carcinoma, N: number, ASD: absolute standardized difference, DM: diabetes mellitus, AMI: acute myocardial infarction

**Table 2:** The events of open angle glaucoma between the two groups

Outcome	Non-NPC (N= 16736)	NPC (N= 4184)	p value
Median follow up months and range (min to max)	85 (38-177)	69 (37-180)	
Person-months	1697960	385200	
Event	513	151	
Incidence density† (95% CI)	0.30(0.28-0.33)	0.39(0.33-0.46)	
Crude HR (95% CI)	Reference	1.303 (1.087-1.563)*	
aHR (95% CI)	Reference	1.293 (1.077-1.551)*	0.0057*

aHR: adjusted hazard ratio which including variables listed in Table 1, CI: confidence interval * denotes significant difference between the two groups † Crude incidence rate, per 1000 person-months.

**Table 3:** Cox regression for estimate the hazard ratio of open angle glaucoma

Parameters	aHR (95% CI)	p value
**NPC**	1.293 (1.077-1.551)	0.0057*
**Year of index (ref=2001-2005)**		
2006-2010	0.854 (0.702-1.038)	0.1125
2011-2015	0.765 (0.530-1.106)	0.1547
**Sex (ref= Female)**		
Male	0.832 (0.705-0.980)	0.0281
**Age (ref=30-40)**		
20-30	0.820 (0.474-1.419)	0.4792
40-50	1.678 (1.191-2.365)	0.0031*
50-60	3.670 (2.634-5.115)	<0.0001*
60-70	5.534 (3.850-7.953)	<0.0001*
70-80	5.692 (3.746-8.648)	<0.0001*
80-100	3.391 (1.601-7.184)	0.0014*
**Co-morbidities**		
Hypertension	1.034 (0.849-1.261)	0.7376
DM	2.132 (1.724-2.635)	<0.0001*
AMI	2.798 (0.673-11.635)	0.1571
Stable CAD	0.845 (0.604-1.181)	0.3234
Hyperlipidemia	0.932 (0.729-1.192)	0.5743
Cerebrovascular disease	1.038 (0.752-1.432)	0.8199
Allergic pulmonary diseases	1.128 (0.894-1.424)	0.3110
Rheumatic disease	1.352 (0.742-2.463)	0.3240
Kidney disease	1.085 (0.716-1.644)	0.7006
Persistent steroid usage	1.890 (1.642-2.176)	<0.0001*

NPC: nasopharyngeal carcinoma, DM: diabetes mellitus, AMI: acute myocardial infarction * denotes significant difference between the two groups

**Table 4:** Subgroup analysis stratified by age and sex for the incidence of open angle glaucoma in nasopharyngeal carcinoma population

Stratification	aHR (95% CI)	p value for interaction
**Sex**		0.5018
Male	1.358(1.073-1.719)	
Female	1.178(0.883-1.571)	
**Age**		0.6131
20-40	1.146(0.631-2.083)	
40-60	1.352(1.047-1.745)	
60-80	1.283(0.956-1.721)	
80-100	0.468(0.042-5.248)	

aHR: adjusted hazard ratio which including variables listed in Table 1, CI: confidence interval

**Table 5:** Treatment for open angle glaucoma patients in study cohorts

Treatments	Non-NPC (N= 513)	NPC (N= 151)
Glaucoma medications		
Alpha agonists	150 (29.2%)	65 (43.0%)
Carbonic anhydrase inhibitors	267 (52.0%)	72 (47.7%)
Beta blockers	363 (70.7%)	115 (76.2%)
Prostaglandin analogues	116 (22.6%)	79 (52.3%)
Glaucoma laser therapy	130 (25.3%)	22 (14.6%)
Glaucoma surgery	21 (4.1%)	0 (0.0%)

N: number, NPC: nasopharyngeal carcinoma

## References

[1] Chua MLK, Wee JTS, Hui EP, Chan ATC (2016). Nasopharyngeal carcinoma. Lancet.

[2] Chang ET, Ye W, Zeng YX, Adami HO (2021). The Evolving Epidemiology of Nasopharyngeal Carcinoma. Cancer Epidemiol Biomarkers Prev.

[3] Chen YP, Chan ATC, Le QT, Blanchard P, Sun Y, Ma J (2019). Nasopharyngeal carcinoma. Lancet.

[4] Jeyakumar A, Brickman TM, Jeyakumar A, Doerr T (2006). Review of nasopharyngeal carcinoma. Ear Nose Throat J.

[5] Lee AWM, Ng WT, Chan JYW, Corry J, Mäkitie A, Mendenhall WM (2019). Management of locally recurrent nasopharyngeal carcinoma. Cancer Treat Rev.

[6] Caponigro F, Longo F, Ionna F, Perri F (2010). Treatment approaches to nasopharyngeal carcinoma: a review. Anticancer Drugs.

[7] Yu B, Lin F, Duan J, Ning H (2022). The influence of marital status on survival in patients with nasopharyngeal carcinoma: A surveillance, epidemiology, and end results database analysis. Medicine (Baltimore).

[8] Wu L, Li C, Pan L (2018). Nasopharyngeal carcinoma: A review of current updates. Exp Ther Med.

[9] Reffai A, Mesmoudi M, Derkaoui T, Ghailani Nourouti N, Barakat A, Sellal N (2021). Epidemiological Profile and Clinicopathological, Therapeutic, and Prognostic Characteristics of Nasopharyngeal Carcinoma in Northern Morocco. Cancer Control.

[10] [Hung SH, Chen PY, Lin HC, Ting J, Chung SD (2014). Association of rhinosinusitis with nasopharyngeal carcinoma: a population-based study. Laryngoscope.

[11] Young YH (2019). Irradiated ears in nasopharyngeal carcinoma survivors: A review. Laryngoscope.

[12] Ku PK, Yuen EH, Cheung DM, Chan BY, Ahuja A, Leung SF (2007). Early swallowing problems in a cohort of patients with nasopharyngeal carcinoma: Symptomatology and videofluoroscopic findings. Laryngoscope.

[13] Liang KL, Jiang RS, Lin JC, Chiu YJ, Shiao JY, Su MC (2009). Central nervous system infection in patients with postirradiated nasopharyngeal carcinoma: a case-controlled study. Am J Rhinol Allergy.

[14] Wong WM, Young SM, Amrith S (2017). Ophthalmic involvement in nasopharyngeal carcinoma. Orbit.

[15] Lee KY, Seah LL, Tow S, Cullen JF, Fong KS (2008). Nasopharyngeal carcinoma with orbital involvement. Ophthalmic Plast Reconstr Surg.

[16] Weinreb RN, Khaw PT (2004). Primary open-angle glaucoma. Lancet.

[17] Weinreb RN, Aung T, Medeiros FA (2014). The pathophysiology and treatment of glaucoma: a review. Jama.

[18] Kang JM, Tanna AP (2021). Glaucoma. Med Clin North Am.

[19] Wey S, Amanullah S, Spaeth GL, Ustaoglu M, Rahmatnejad K, Katz LJ (2019). Is primary open-angle glaucoma an ocular manifestation of systemic disease?. Graefes Arch Clin Exp Ophthalmol.

[20] Lee SH, Kim GA, Lee W, Bae HW, Seong GJ, Kim CY (2017). Vascular and metabolic comorbidities in open-angle glaucoma with low- and high-teen intraocular pressure: a cross-sectional study from South Korea. Acta Ophthalmol.

[21] Lee WJ, Jeoung JW, Na KI, Kim YK, Kim CY, Park KH (2018). Relationship Between Open-angle Glaucoma and Stroke: A 2010 to 2012 Korea National Health and Nutrition Examination Survey. J Glaucoma.

[22] Chen YY, Hu HY, Chu D, Chen HH, Chang CK, Chou P (2016). Patients with Primary Open-Angle Glaucoma May Develop Ischemic Heart Disease More Often than Those without Glaucoma: An 11-Year Population-Based Cohort Study. PLoS One.

[23] Lee CY, Liu CH, Chen HC, Sun CC, Yao YP, Chao SC (2019). Correlation between Basal Macular Circulation and Following Glaucomatous Damage in Progressed High-Tension and Normal-Tension Glaucoma. Ophthalmic Res.

[24] Ramtohul P, Beylerian M, Dambricourt L, Matonti F, Denis D (2019). Secondary Congenital Glaucoma Associated With Retro-orbital Infantile Hemangioma: A Masquerade Syndrome. J Glaucoma.

[25] Sears NC, Singh A, Singh AD (2016). Ocular Adnexal Lymphoma Presenting as Refractory Unilateral Open-angle Glaucoma. J Glaucoma.

[26] Chau SF, Wu PH, Sun CC, Huang JY, Nien CW, Yang SF (2019). The Development of Glaucoma after Surgery-Indicated Chronic Rhinosinusitis: A Population-Based Cohort Study. Int J Environ Res Public Health.

[27] Shin SC, Hong SL, Lee CH, Cho KS (2016). Orbital metastasis as the primary presentation of nasopharyngeal carcinoma. Braz J Otorhinolaryngol.

[28] Wahab Z, Tai E, Wan Hitam WH, Sonny Teo KS (2021). Corticosteroid Therapy in Optic Neuropathy Secondary to Nasopharyngeal Carcinoma. Cureus.

[29] Hsu WM, Wang AG (2004). Nasopharyngeal carcinoma with orbital invasion. Eye (Lond).

[30] Founti P, Coleman AL, Wilson MR, Yu F, Harris A, Pappas T (2021). Twelve-Year Incidence of Open-angle Glaucoma: The Thessaloniki Eye Study. J Glaucoma.

[31] Pan CW, Yang WY, Hu DN, Xu JG, Niu ZQ, Yuan YS (2017). Longitudinal Cohort Study on the Incidence of Primary Open-Angle Glaucoma in Bai Chinese. Am J Ophthalmol.

[32] Leske MC (2007). Open-angle glaucoma - an epidemiologic overview. Ophthalmic Epidemiol.

[33] Quigley HA, Broman AT (2006). The number of people with glaucoma worldwide in 2010 and 2020. Br J Ophthalmol.

[34] Quigley HA (2011). Glaucoma. Lancet.

[35] Distelhorst JS, Hughes GM (2003). Open-angle glaucoma. Am Fam Physician.

[36] Mwanza JC, Tulenko SE, Barton K, Herndon LW, Mathenge E, Hall A (2019). Eight-Year Incidence of Open-Angle Glaucoma in the Tema Eye Survey. Ophthalmology.

[37] Zhou M, Wang W, Huang W, Zhang X (2014). Diabetes mellitus as a risk factor for open-angle glaucoma: a systematic review and meta-analysis. PLoS One.

[38] Li Y, Mitchell W, Elze T, Zebardast N (2021). Association Between Diabetes, Diabetic Retinopathy, and Glaucoma. Curr Diab Rep.

[39] Havens SJ, Gulati V (2016). Neovascular Glaucoma. Dev Ophthalmol.

[40] Lin KT, Lee SY, Liu SC, Tsao CC, Hsu SD, Chien WC (2020). Risk of ocular complications following radiation therapy in patients with nasopharyngeal carcinoma. Laryngoscope.

[41] Brook I (2020). Late side effects of radiation treatment for head and neck cancer. Radiat Oncol J.

